# Development of a practical framework for sustainable surveillance and control of ticks and tick-borne diseases in Africa

**DOI:** 10.14202/vetworld.2020.1910-1921

**Published:** 2020-09-19

**Authors:** Felix Nchu, Nkululeko Nyangiwe, Dennis Muhanguzi, Jahashi Nzalawahe, Yakob Petro Nagagi, George Msalya, Natala Audu Joseph, Esther Gwae Kimaro, Margaret Mollel, Violet Temba, Difo Voukang Harouna

**Affiliations:** 1 Department of Horticultural Sciences, Cape Peninsula University of Technology, Bellville, Symphony Way, Bellville, Cape Town, 7535, South Africa; 2 Department of Rural Development and Agrarian Reform, Döhne Agricultural Development Institute, Private Bag X15, Stutterheim 4930, South Africa; 3 Department of Conservation Ecology and Entomology, University of Stellenbosch, Stellenbosch 7602, South Africa; 4 Department of Biomolecular and Biolaboratory Sciences, College of Veterinary Medicine Animal Resources and Biosecurity, Makerere University, P.O. Box 7062, Kampala, Uganda; 5 Department of Veterinary Microbiology, Parasitology and Biotechnology, College of Veterinary Medicine and Biomedical Sciences, Sokoine University of Agriculture, P. O. Box 3019, Chuo Kikuu, Morogoro, Tanzania; 6 Division of Livestock and Human Diseases Vector Control, Tropical Pesticides Research Institute, P.O. Box 3024, Arusha, Tanzania; 7 Department of Animal, Aquaculture and Range Sciences, College of Agriculture, Sokoine University of Agriculture, P. O. Box 3004, Chuo Kikuu, Morogoro, Tanzania; 8 Laboratory of Animal Breeding and Genetics, Kagoshima University, 1-21-24 Korimoto, Kagoshima 890-0065, Japan; 9 Department of Veterinary Parasitology, Faculty of Veterinary Medicine, Ahmadu Bello University, Zaria, Nigeria; 10 Department of Food Biotechnology and Nutritional Sciences, Nelson Mandela African Institution of Science and Technology, P. O. Box 447, Arusha, Tanzania

**Keywords:** Africa, consortium, ticks, tick-borne diseases

## Abstract

A workshop on ticks and tick-borne diseases (T&TBDs) was held on June 25 and 26, 2019, at the Tropical Pesticides Research Institute, Division of Livestock and Human Diseases Vector Control, Arusha, Tanzania. The objectives of the workshop were to discuss the current situation and to formulate actionable strategies to improve surveillance and control of T&TBDs in Africa. The workshop was funded by the National Research Foundation and the Cape Peninsula University of Technology and attended by livestock health providers, farmers, and researchers from East, West, and Southern African countries. During the workshop, experts presented recent surveillance data focused on T&TBDs; participants discussed research opportunities and community engagement. The primary outcome of the workshop was the creation of a new research consortium known as The African Consortium for T&TBDs. The consortium is intended to function as a community for researchers, students, farmers, policymakers, extension workers, and community members who are interested in the advancement of T&TBD control. The consortium will engage in research activities that focus on comprehensive surveillance of T&TBDs, developing tick acaricide resistance, alternative tick control programs, and policy development and education. These areas were identified as top priorities to be developed to improve T&TBD control on the continent.

## Introduction

Ticks are second only to mosquitoes as vectors of human and animal pathogens [1]. Tick infestations can have devastating effects on human health as well as on livestock and hence the livelihoods of livestock farmers [2]. Indisputably, the burden of tick and tick-borne diseases (T&TBDs) on the economies and livelihoods of all those involved in the livestock industry in Africa remains significant [3,4]. Several reasons have been put forward to explain the consistent and unremitting increase in the incidence of T&TBDs. These include poor veterinary and healthcare services, inadequate monitoring and surveillance programs targeting T&TBDs, deforestation and human encroachment on wildlife habitats, tick resistance to acaricides, and climate change. These factors have also been identified to be among the most likely drivers of emerging zoonotic diseases [5].

Efforts to curb T&TBDs are intensifying worldwide. Monitoring and surveillance programs are among the most reliable tools for the sustainable management of T&TBDs. When used appropriately, they can provide targeted control interventions, enable timely detection of high-risk areas and emerging acaricide resistance, reduce the misuse of acaricides, document the movement of ticks on translocated livestock, facilitate the development of effective policies, and provide longterm and far-reaching datasets that predict future disease outbreaks and risk assessment. Despite the well-known benefits associated with effective tick surveillance and monitoring, success stories focused on these practices in Africa are very few. Active or even passive country-wide T&TBDs surveillance programs have not been conducted in any African countries over the last few decades. Furthermore, there are no inter-territorial T&TBD sampling protocols that facilitate data comparison and risk assessment. Many tick surveys still rely on morpho-taxonomic keys that are both cumbersome and inaccurate, especially when used to identify damaged, engorged, and sub-adult stages of ticks from species that are difficult to distinguish from one another on a purely morphologic basis [6]. Given these challenges, we organized a T&TBDs workshop at the Tropical Pesticides Research Institute in Arusha, Tanzania. We intended to discuss high-impact research approaches and programs that might ultimately serve to tackle some of the pervasive challenges that ultimately hinder the effective T&TBD management in East, West, and Southern African countries. As such, the objectives of the workshop were to discuss the current situation concerning surveillance of T&TBDs in selected African countries, to formulate actionable strategies within an adaptive framework, and to improve the monitoring and control of T&TBDs in East, West, and Southern African countries.

## Monitoring and Surveillance of T&TBDs in Africa

Extensive and comprehensive T&TBD surveillances can shed light on the seasonal, temporal, and spatial variability of ticks and tick-borne hemoparasites. Surveillance approaches that are often applied when sampling ticks include both active and passive tick surveillance. Active tick surveillance involves dragging, flagging, semiochemical-based trapping, and live animal capture. Passive tick surveillance, which is generally less costly, relies on farmers and volunteers who submit ticks for identification and pathogen screening [7]. Combined use of both surveillance approaches may be more effective in providing information on tick abundance in a given habitat. Recently, Fryxell and Vogt [8] demonstrated that collaborative tick surveillance programs involving academia and government partnerships might be improved to generate useful data, including pathogen detection, revisiting sites of detection, and hence providing continuous updates of tick encounters. In Africa, long-term and broad-based T&TBD surveillance programs are rare. Furthermore, a handful of T&TBDs surveillance programs, both past and present, have generated only limited data that are relevant at the national and regional levels; this may be because most surveys are restricted to particular geographic areas and/or brief periods of time. However, scientific evidence suggests that longterm passive tick surveillance is a meaningful and credible approach that might be used to explore the ecology of both common and rare tick species [7]. Although community members in both the United States and the United Kingdom provide significant contributions toward this effort by participating in passive tick surveillance, passive sampling, and community involvement in Africa is rare. However, most researchers believed that community-based tick sampling in Africa is feasible and can be achieved. Small-scale livestock farmers in Africa often live in poor rural areas. The farmers in these communities communally graze their livestock and have strong cultural and social bonds; they may be highly motivated partners in an appropriately designed T&TBD surveillance and control project.

## Highlights of the Gaps in T&TBDs Surveillance and Control Programs in Africa: Individual Country Reports

Tick population dynamics have revealed critical shifts in the compositions of tick communities; there are many reports of cases in which exotic tick species invade new territories and replace some specific of indigenous ticks. This has largely been due to the translocation of exotic ticks from their native lands to new habitats through imported livestock. These observations, together with other factors, including inadequate veterinary and healthcare services and T&TBD monitoring and surveillance, land use changes such as deforestation and human encroachment on wildlife habitats, tick resistance to acaricides, and climate change, all result in rapid expansion of the tick population and hence TBDs [9-11]. To cope up with the challenge of monitoring and managing T&TBDs associated with livestock and human health, this workshop was organized to review existing T&TBDs surveillance and control programs and to identify gaps in these programs in selected East, West, and Southern African territories, including Uganda, Tanzania, Nigeria, and South Africa. The participants from these countries discussed individual country reports focused on the current state of T&TBDs. Based on the reports from the individual countries, it became apparent that there were few in-depth and comprehensive studies of T&TBDs surveillance in these African countries. Consequently, invasive tick species and zoonotic diseases are spreading rapidly across the continent.

### Uganda

Cross-sectional and focal tick surveys have revealed that *Rhipicephalus* spp. are the most abundant tick species in Uganda [12-14] (Table-1). The few focal studies that have been conducted indicate that tick density varies greatly between different agroecological zones in Uganda, with the highest tick density recorded in the Lake Kyoga and Lake Victoria Crescent districts. However, there have been no tick surveys across 11 agroecological zones and during both the wet and dry seasons. Similar to what we learned regarding tick surveys, tick-borne hemoparasites (TBPs) surveys have all been cross-sectional and focal. These studies have identified *Theileria parva*, *Anaplasma marginale*, *Ehrlichia ruminantium*, *Babesia bovis*, and *B. bigemina* as among the most economically important of the circulating TBPs [14-18,19] (Table-1).

**Table-1:** Predominant species of ticks and tick-borne pathogens surveyed in various locations in South Africa, Nigeria, Tanzania and Uganda.

Predominant tick species/Tick-borne pathogens/diseases	Hosts or vegetation	Area surveyed	Year	References
Predominant tick species in South Africa				
*Amblyomma hebraeum*	Cattle	Eastern Cape Province (north-eastern regions)	2009; 2011	[34,35]
*Rhipicephalus microplus*	Goats			
*Rhipicephalus appendiculatus*				
*Rhipicephalus evertsi evertsi*				
*Rhipicephalus decoloratus*				
*Rhipicephalus follis*				
*Rhipicephalus evertsi evertsi*	Cattle,	Northwest Province (North–eastern, Central and Western regions)	2011	[43]
*Hyalomma rufipes*	Goats			
*Amblyomma hebraeum*	Sheep			
*Rhipicephalus decoloratus*				
*Rhipicephalus appendiculatus*				
*Hyalomma truncatum*				
*Rhipicephalus microplus*				
*Rhipicephalus evertsi mimeticus*				
*Rhipicephalus simus*				
*Rhipicephalus microplus*	Cattle	Limpopo Province (Soutpansberg and Thabazimbi district)	2004; 2013	[37,44]
*Rhipicephalus decoloratus*	Vegetation			
*Amblyomma hebraeum*				
*Rhipicephalus appendiculatus*				
*Rhipicephalus evertsi evertsi*				
*Hyalomma rufipes*				
*Rhipicephalus zambeziensis*				
*Amblyomma hebraeum*	Donkeys, Horses	Gauteng Province (Pretoria)	2017	[45]
*Rhipicephalus evertsi evertsi*				
*Hyalomma rufipes*				
*Rhipicephalus microplus*				
*Rhipicephalus decoloratus*				
*Rhipicephalus appendiculatus*				
*Hyalomma truncatum*				
*Rhipicephalus microplus*	Cattle, donkeys, horses, dogs	Northern Cape Province (Northern-eastern regions)	2017; 2010	[32,44,46]
*Rhipicephalus decoloratus*				
*Margaropus winthemi*				
*Rhipicephalus evertsi evertsi*				
*Rhipicephalus gertrudae*				
*Ixodes rubicundus*				
*Rhipicephalus sanguineus*				
*Hyalomma truncatum*				
*Rhipicephalus microplus*	Cattle, donkeys, goats, horses	Mpumalanga	2002; 2015; 2017	[45,47,48]
*Amblyomma hebraeum*				
*Rhipicephalus decoloratus*				
*Rhipicephalus appendiculatus*				
*Rhipicephalus evertsi evertsi*				
*Rhipicephalus simus*				
*Rhipicephalus zambeziensis*				
*Amblyomma hebraeum*	Cattle, goats, sheep	KwaZulu Natal (Umsinga)	2015	[39]
*Rhipicephalus decoloratus*				
*Rhipicephalus appendiculatus*				
*Rhipicephalus evertsi evertsi*				
*Rhipicephalus decoloratus*	Cattle,	Free State Province (northwest, south-west and south of the province)	2015	[31]
*Rhipicephalus appendiculatus*	Sheep			
*Ixodes rubicundus*	Eland			
*Amblyomma hebraeum*	Gemsbok			
*Rhipicephalus microplus*	White rhinoceroses			
*Rhipicephalus evertsi mimeticus*				
*Rhipicephalus decoloratus*	Cattle,	Western Cape	2017	[31]
*Rhipicephalus appendiculatus*	Donkeys,			
*Rhipicephalus microplus*	Horses			
*Margaropus winthemi*				
*Rhipicephalus evertsi evertsi*				
*Rhipicephalus gertrudae*
Tick-borne pathogens/diseases in South Africa				
*Babesia bigemina*	Cattle	Eastern Free State of South Africa	1998-2000	[38,49]
*Anaplasma* spp.	Sheep and goat			
*Babesia* spp., *Theileria* spp.,* Anaplasma marginale, Rickettsia* spp.,* Ehrlichia ruminantium* and *Coxiella burnetii*	Tick	KwaZulu-Natal, Free State and Eastern Cape of South Africa	2018	[40]
*Theileria* spp., *Anaplasma ovis* and *Ehrlichia ruminantium*	sheep and goat	Free State and KwaZulu-Natal provinces, South Africa	2018	[41]
*Anaplasma marginale*	Wildlife (*Oryx*	Free State Province, South Africa	2006	[50]
*Theileria* spp.	*gazella gazella*)			
*Theileria* spp.	Tick			
*Theileria separate*				
*Anaplasma bovis*				
*Rickettsia africae*	Human	Swedish tourists who visited South Africa	2004; 2015-2016	[42,51]
*Rickettsia mongolotimonae*	Human	Near Ellisras, Limpopo Northern Province	2002	[52]
*Ehrlichia chaffeensis, Ehrlichia canis,* *Ehrlichia muris, Ehrlichia* spp. UFMG-EV and *Ehrlichia* spp. UFMT	Ticks on domesticated	Chris Hani District Municipality, Eastern Cape Province	2016	[53]
	cattle, sheep and goats) and horses			
*Babesia bovis, Babesia bigemina* and *Anaplasma marginale*	Cattle	Magwiji, Ukhahlamba district, and Cala, Chris Hani district communal rangelands of the Eastern Cape Province	2007-2008	[54]
Predominant tick species in Nigeria				
*Rhipicephalus* spp.	Cattle	Vom, Plateau State	1986	[25]
*Rhipicephalus* spp.				
*Rhipicephalus* spp.	Cattle	Mokwa, Niger State	1986	[25]
*Hyalomma* spp.				
*Amblyomma* spp.				
*Rhipicephalus* spp.	Dog	Makurdi, Benue State	2007	[55]
*Amblyomma* spp.				
*Rhipicephalus sanguineus*	Dog	Makurdi Benue State	2008	[56]
*Amblyomma variegatum*	Cattle	Plateau State	2017	[27]
*Rhipicephalus sanguineus*	Dog	Jos Plateau State	2018	[57]
*Rhipicephalus decoloratus*				
*Haemaphysalis leachii*				
*Rhipicephalus decoloratus*	Cattle	Lafia Nasarawa State	2019	[58]
*Amblyomma variegatum*				
*Hyalomma rufipes*				
*Hyalomma spp*	Cattle	Runka, Katsina State	1974	[59]
*Amblyomma variegatum*				
*Rhipicephalus decoloratus*				
*Amblyomma variegatum*	Cattle	Samaru, Kaduna State	1974	[59]
*Rhipicephalus decoloratus*				
*Rhipicephalus evertsi*	Horse	Zaria, Kaduna State	2002	[60]
*Amblyomma variegatum*				
*Argas persicus*	Birds	Sokoto, Sokoto State	2008	[61]
*Argas walkarae*				
*Ornithodoros moubata*				
*Hyalomma dromedarii*	Camel	Sokoto, Sokoto State	2015	[62]
*Hyalomma rufipes*				
*Hyalomma impeltatum*				
*Hyalomma truncatum*				
*Rhipicephalus simus*	Cattle	Zaria, Kaduna State	2019	[28]
*Rhipicephalus sanguineus*				
*Rhipicephalus decoloratus*				
*Amblyomma variegatum*				
*Rhipicephalus microplus*				
*Amblyomma spp*	Cattle	Mambila, Taraba State	1986	[25]
*Amblyomma variegatum.*	Cattle	Yobe and Borno State	2011	[63]
*Hyalomma* spp.				
*Rhipicephalus* spp.				
*Dermacentor* spp.				
*Rhipicephalus* spp.	Dog	Maiduguri, Borno State	2014	[64]
*Rhipicephalus* spp.				
*Amblyomma variegatum*	Cattle	Maiduguri, Borno State	2017	[65]
*Rhipicephalus sanguineus sensu lato*				
*Rhipicephalus) decoloratus*				
*Hyalomma truncatum*				
*Rhipicephalus* spp.	Cattle	Ogun State	2013	[26]
*Rhipicephalus* spp.				
*Amblyomma* spp.				
*Rhipicephalus sanguineus*	Dog	Ogun, State	2018	[66]
*Haemaphysalis leachi leachi*				
*Amblyomma variegatum*
Tick-borne pathogens/diseases in Nigeria				
*Theileria velifera*	Cattle	Vom, Plateau State	1986	[25]
*Theileria mutans*	Cattle			
*Theileria mutans,*	Cattle	Mokwa, Niger State	1986	[25]
*Anaplasma marginale*	Cattle			
*Babesia canis*	Dog	Makurdi, Benue State	2007	[55]
*Hepatozoon canis*	Dog and Tick (DNA)	Plateau State	2012	[30]
*Ehrlichia canis*				
*Rickettsia spp.*				
*Babesia rossi*				
*Anaplasma platys*				
*Babesia bigemina*	Tick of Cattle and Dog	Jos, Plateau State	2012	[29]
*Babesia divergens*				
*Anaplasma marginale*				
*Rickettsia africae*				
*Theileria mutans*	Cattle	Plateau State	2016	[67]
*Theileria velifera*	Cattle			
*Theileria taurotragi*	Cattle			
*Anaplasma marginale*	Cattle			
*Ehrlichia ruminantium*	Cattle			
*Anaplasma* spp. *Babesia* spp.	Goat and Sheep	Makurdi, Benue State	2018	[68]
*Babesia bovis*	Cattle	Gidan Jaja, Katsina	1986	[25]
*Anaplasma marginale*	Cattle	State		
*Babesia canis*	Dog	Zaria, Kaduna State	2013	[69]
*Babesia perronatoi*	Pig	Zaria, Kaduna State	2014	[70]
*Anaplasma phagocytophilum*	Cattle	Zaria, Kaduna State	2019	[28]
*Anaplasma ovis*	Sheep and Goat	Maiduguri, Borno State	2017	[71]
*Babesia ovis*	Sheep and Goat			
*Theileria mutans*	Cattle	Fashola, Oyo State	1987	[25]
*Theileria velifera*	Cattle			
*Theileria mutans*	Cattle	Akunnu, Ondo State	1987	[25]
*Rickettsia* spp.	Cattle	Ibadan, Oyo State	2012	[72]
*Coxiella burnetii*				
*Anaplasma* spp.				
*Ehrlichia* spp.				
*Babesia* spp.	Cattle	Ogun State	2013	[26]
*Anaplasma* and * Babesia*	Cattle
Predominant tick species in Tanzania				
*Rhipicephalus appendiculatus* and *Amblyomma variegatum*	Cattle	Shinyanga, Southern Highlands, Tabora, Arusha and Dar es Salaam	1973-1976	[21]
*Rhipicephalus appendiculatus, Rhipicephalus evertsi, Rhipicephalus kochi* and *Haemaphysalis leachii*	Cattle, Goats, Sheep and Rodents	Lower Kihansi (Iringa and Morogoro)	2000	[73]
*Rhipicephalus appendiculatus, Amblyomma* spp. (*A. variegatum, A. gemma* and * Amblyomma lepidum)* and *Rhipicephalus* spp. (*R. decoloratus* and *R. microplus*)	Cattle	Lake zone (Mwanza, Kagera, Mara and Shinyanga)	1998-2001	[22]
		Southern Highlands (Iringa and Mbeya)		
		Southern zone (Mtwara, Ruvuma and Rukwa)		
		Western zone (Kigoma and Tabora)		
		Central zone (Dodoma and Singida)		
*Rhipicephalus appendiculatus, Rhipicephalus evertsi, Amblyomma variegatum, Hyalomma* spp. and *Rhipicephalus decoloratus*	Cattle	Ngorongoro district	2004	[74]
*Amblyomma gemma, Amblyomma variegatum, Rhipicephalus pulchellus* and *Hyalomma impeltatum*)	Cattle, Buffalo, Bush buck, Bush pig, Eland, Leopard and Warthog	Iringa and Maswa	2012	[75]
*Amblyomma variegatum, Rhipicephalus microplus, Rhipicephalus evertsi and Rhipicephalus appendiculatus*	Cattle	Rufiji district	2009, 2011 and 2012	[24]
*Amblyomma variegatum* and *R. appendiculatus*	Cattle	Mara region	2015	[76]
*Amblyomma lepidum, A. variegatum, Rhipicephalus microplus, Hyalomma rufipes* and *Rhipicephalus appendiculatus*	Cattle	Singida region	2015	[76]
*Hyalomma rufipes, Rhipicephalus evertsi* and *Rhipicephalus microplus*	Cattle	Mbeya region	2015	[76]
Tick-borne pathogens/diseases in Tanzania				
*Theileria* spp., *Anaplasma* spp. and *Babesia* spp. (37.1%)	Cattle	Same district	2013-2014	[77]
*Anaplasma* spp., *Ehrlichia* spp., *Babesia* spp., *Theileria* spp. and *Rickettsia* spp.	Tick	Maswa and Iringa	2012	[75]
*Theileria* spp., *Babesia bigemina, Anaplasma marginale, Ehrlichia ruminantium* and *Babesia bovis*	Cattle	Pemba Island	2017	[41]
*Anaplasma marginale*	Ticks	Ngorongoro crater	2001 - 2005	[78]
*Anaplasma bovis, Babesia equi, Theileria buffeli,* and *Theileria parva*	Ticks	Ngorongoro crater	2001 - 2005	[79]
Predominant tick species in Uganda				
*Rhipicephalus* spp.* (R. appendiculatus,* *R. evertsi evertsi, R. microplus, R. decoloratus, R. afranicus, R. pulchellus,* *R. simus, R. sanguineus, R. turanicus* and *R. muhsamae)* and *Amblyomma spp.* *(A. lepidum, A. variegatum, A. cohaerens, Amblyomma gemma,* and *A. paulopunctatum)*	Cattle	In isolated districts of south-western, south-eastern Uganda and north-western regions of Uganda	2008-2020	[13,80-84]
Tick-borne pathogens/diseases in Uganda				
*Theileria* spp. (*T. parva, T. mutans* *T. taurotragi, T. vilifera, T. buffeli, T.* spp. [sable], *T.* spp. [buffalo] and *T. bicornis), Babesia* spp. (*B. bovis, B. bigemina* and *B. vogelli)* *Anaplasma* spp. *(A. marginale,* *A. centrale and A. phagocytophilum), Rickettsia/Ehrlichia* spp. (*E. ruminantium,* *E. africae, E. ovina/canis, E.* spp. [*omatjenne*]) and *Coxiella burnetii*	Cattle	In isolated districts of south-western, south-eastern Uganda and north-western and central regions of Uganda	2004-2020	[81,85-89]

### Tanzania

The most comprehensive survey of ticks in Tanzania was conducted in the 1950s and 1960s [20]. Since that time, two additional comprehensive studies revealed marked expansion of tick species, most notably *Rhipicephalus microplus* and *Rhipicephalus appendiculatus*, in areas previously not occupied by these species [21,22] (Table-1). Another study based on Geographical Information System (GIS) collected on an extensive field survey for *R. appendiculatus and R. pravus*, as well as *for Amblyomma* species in cattle rearing areas of Tanzania between July 1998 and March 2001, found that cattle density influenced the distribution of *A. variegatum* and, to a certain extent, of *A. lepidum*, but had no appreciable influence on the distribution of other ticks studied [23]. The *R. microplus* is nearly dominant in this new habitat and has completely displaced *Rhipicephalus decoloratus* in regions where they previously coexisted [22]. Furthermore, the previous studies reported widespread distribution of both *Amblyomma* (especially *Amblyomma variegatum*) and *Rhipicephalus* spp., with *R. appendiculatus* identified as the most abundant species in both the northern (Arusha and Manyara regions) and southern (districts of the Mtwara and Rukwa regions) agroecological zones [22]. The migration and re-settlement of livestock farmers who are searching for ample grazing lands for their animals have contributed to the spread of economically important tick species. A survey conducted in the new livestock farming region in Rufiji, on the coastal region of Tanzania, has revealed that various tick species are widely established in this area, with the highest distribution observed for *A. variegatum* and *R. microplus* [24]. The widespread distribution of *Amblyomma* spp. and *R. appendiculatus* has contributed to the development of major economic threats to the livestock industry in this country, including heartwater, anaplasmosis, East Coast fever (ECF), and, to some extent, babesiosis (Table-1).

### Nigeria

Nigeria is divided into six geopolitical regions, namely, North-Central (NC), North-West (NW), North-East (NE), South-West (SW), South-East (SE), and South-South (SS). T&TBDs surveillance was conducted somewhat more frequently in the Northern parts of the country in contrast to Southern Nigeria [25,26]. Most reports on T&TBD surveillance came from the states of Plateau (NC), Benue (NC), Kaduna (NW), and Borno (NE) and covered the years 1974-2019 [25,27,29]. The most comprehensive of these studies, which focused on T&TBD pathogens, was conducted over 30 years ago; this study relied on morphology, cytology, and serology as diagnostic tools [25]. The major tick populations encountered in Nigeria included *A. variegatum*, *R. decoloratus*, *Rhipicephalus sanguineus*, *Rhipicephalus simus*, and *Hyalomma* spp. [25,26] (Table-1). Notably, *R. microplus* was also recorded in a recent study in Zaria [28]. The genera of tick-borne pathogens of prominence include *Babesia* spp., *Anaplasma* spp., *Theileria* spp., *Hepatozoon* spp., and *Ehrlichia* spp. [25,30] (Table-1). Studies that include molecular surveillance of T&TBDs are quite rare in Nigeria [27]. A means for monitoring T&TBDs in the six geopolitical zones in Nigeria using modern molecular surveillance techniques is necessary. These studies would provide critical baseline information and may also serve to validate or invalidate earlier studies based on primarily morphological criteria. Furthermore, given the trans-boundary movement of animals, long-term monitoring of T&TBDs will facilitate the timely detection of ticks that are introduced into Nigeria from other countries and will permit timely control strategies to be put in place.

### South Africa

The geographical distribution of several tick species is currently changing in South Africa; ticks of the genera *Amblyomma* and *Rhipicephalus* have recently expanded their distributional ranges [31-33]. In many parts of the country, *R. microplus* is in the process of invading localities where the native tick *R. decoloratus* remains to be the prevalent species [32,34-37] (Table-1). A recent survey of TBDs in South Africa concentrated on the Eastern Cape, Free State, and the KwaZulu-Natal Provinces [38-41]. The predominant TBD pathogens identified among livestock in South Africa belong to the genera *Babesia*, *Theileria*, *Anaplasma*, and *Ehrlichia* (Table-1) [13,21-32,34,35,37-89]. Notably, land use, as well as habitat and climate change, may increase the frequency of interactions and sharing of tick-borne pathogens between humans, wildlife, and livestock [90,91]; these host– pathogen–environment interactions have not been explored in South Africa.

## Alternative Methods of Controlling Ticks

At this workshop, three such methods were discussed; these include ethnoveterinary practices, antitick vaccines, and livestock breeding for T&TBDs resistance.

## Ethnoveterinary Practices in Africa

Ethnoveterinary medicine and practices are widespread across Africa and are typically preferred by small-scale farmers in rural areas, as they are based on traditional knowledge that is transferred from generation to generation. Cultural practices associated with ethnoveterinary practices are not properly documented and are disappearing quickly. There are numerous documented anti-tick ethnoveterinary plants found in Southern, East, West, Central, and North Africa. In East Africa, of the 47 plant species have been documented as useful for tick control, only 14 (30%) have been scientifically validated. Similarly, in Southern Africa, only nine of 36 (25%) of the plants traditionally used to combat ticks have undergone scientific validation [92]. A similar situation exists in West Africa, where only three of the 13 (23%) of the plant species used to treat TBDs have been validated experimentally. As such, experiments aimed at validating the antitick activities of a variety of ethnoveterinary plants should be performed, and documentation of ethnoveterinary practices must be provided. Current research opportunities in ethnoveterinary medicine include the evaluation and validation of traditional claims, isolation of biologically active compounds from these plants, optimization of secondary metabolite production, development of herbal-based antitick products, and documentation and standardization of ethnoveterinary plant species.

## Anti-tick Vaccines

Vaccination of livestock with immunologically active tick extracts might serve to generate antibodies in the vertebrate hosts; when ticks feed on animals with serum anti-tick antibodies, these may disrupt essential pathways, thereby reducing tick survival. There has been renewed interest in this rather old approach; current research suggests that this strategy might be beneficial for promoting tick control [93,94]. Renewed interest in this strategy has been triggered by the rising trend in acaricide resistance together with advancements in bioinformatics. Updates from Uganda indicated that the Molecular and Computational Biology Research group of Dr. Muhanguzi Dennis at the College of Veterinary Medicine, Animal Resources and Biosecurity are conducting both protein [84] and transcriptome/proteome studies *in silico* to identify candidate peptides to be included in future anti-tick vaccine pipelines. The AfriCoTT consortium provides an important opportunity to extend this effort to include all participating countries and institutions to accelerate the entry of any peptides identified into *in vitro* and *in vivo* testing. Given the use of funds leveraged through this consortium, these efforts may be scaled up to include the identification of new targets for acaricide to expand their therapeutic range. This is especially important because some of the currently available acaricides have been overused, leading to the development of acaricide-resistant tick populations.

## Breeding Livestock for Tick Resistance

Indigenous cattle on the African continent are believed to possess an inherent capacity to withstand diseases, heat stress, and food scarcity [95-98]. For example, West African N’Dama cattle are tolerant of trypanosomiasis [99]. In Kenya, the Small East African zebu were reported to be resistant to *R. appendiculatus* ticks [100], whereas in Tanzania, preliminary results have revealed that the Tanzania shorthorn zebu may be tolerant of ticks as well as to ECF [101,102]. However, the scientific basis of these findings has not been studied explicitly or described extensively in the scientific literature. Further studies that are focused on assessments of the level of tolerance observed in various breeds and populations of cattle in Africa are required. This will provide an avenue in which highly tolerant animals cross-bred to conserve this feature as well as to capitalize on their unique resistance/tolerance to TBDs. The consortium (AfriCoTT) can be a platform for researchers in the participating countries and for prospective members for sharing information regarding verification of the genetic potential of various breeds and animal populations that might be utilized in these programs.

## The Way Forward

Approaches to integrated tick control that incorporate traditional cultural practices, education, and partnership within affected communities would certainly improve the efficacy of current tick management programs. As a group, the participants proposed a participatory approach that involves students, researchers, government agencies, and communities to enhance surveillance for T&TBDs, encourage multidisciplinary participatory research, and promote a means to share limited resources and knowledge. Multiple tick sampling approaches, including both passive and active approaches, might be the best toward achieving the goal of tick surveillance and control. Cultivated pastures were proposed as a means for improving security and minimizing the spread of ticks; however, some participants felt that their establishment and maintenance would be overly expensive. Participants identified that a platform where researchers could share ideas, expertise, and resources should be established. A practice community that is open to researchers, policymakers, communities, livestock businesses, pharmaceutical companies, and farmers was recommended by the meeting participants. Subsequently, the participants at the meeting conceived the consortium (AfriCoTT) whose mission would be to conduct survey and research on T&TBDs in Africa. The overarching goal of AfriCoTT is to improve the surveillance and management of T&TBDs in Africa through rigorous and high-impact research. The following thematic areas were developed, focused on the need to:

- Establish national T&TBD survey programs initially in at least ten African countries;
- Develop national acaricide susceptibility profiles;
- Conduct ecological investigations on T&TBDs both regionally and nationally;
- Conduct research on innovative and culturally acceptable tick control options;
- Implement social-based thematic areas that will have an impact on T&TBD management, including attitudes, practice, and cultures; and
- Influence policy development at both national and regional levels.

## Anticipated Impacts

- Development of five major interdisciplinary research themes.
- Publication and dissemination of research outputs.
- Collaboration with a wide range of partners at local, regional, and international levels.
- Training and mentoring of students.
- Provision of a platform for sharing of ideas and for promoting collaborative work.
- Contribution to the efforts required for ongoing management of T&TBDs in Africa.
- Establishment of strategic partnerships with universities, research centers, communities, farmers, volunteers, policy makers, and students.

## Action Plan

The work of the consortium will continue over 5 years. Activities are organized into three levels over 5 years.

Level 1: Initially, the consortium will focus on recruiting participants from each country. Participants will be tasked with developing research projects that focus on surveillance of T&TBDs and acaricide resistance. The participants will also develop and manage local T&TBD databases, recruit students, and volunteers in the communities, and collaborate with their counterparts in other African countries.

Level 2: Research projects will focus on surveys of ethnoveterinary plants and practices and also on other alternative tick control approaches, including animal breeding and immunization strategies. Surveillance data will be collected for use in ecological investigations of T&TBDs.

Level 3: Exploring the social and cultural aspects associated with T&TBD management will be emphasized. Participants will be involved in developing policies and strategies for containment and surveillance of T&TBDs.

## Authors’ Contributions

FN, NN, DM, JN, YPN, GM, NAJ, EGK, MM, VT, and DVH conceptualized and designed, drafted, revised, and finalized the report. All authors read and approved the final meeting report.
